# Apicomplexans in Goat: Prevalence of *Neospora caninum*, *Toxoplasma gondii*, *Cryptosporidium* spp., *Eimeria* spp. and Risk Factors in Farms from Ecuador

**DOI:** 10.3390/ani12172224

**Published:** 2022-08-29

**Authors:** Kevin Celi, Lucía Guzmán, Catalina Rey-Valeirón

**Affiliations:** 1 Departamento de Ciencias Biológicas y Agropecuarias, Facultad de Ciencias Exactas y Naturales, Maestría en Biotecnología Agropecuaria, Universidad Técnica Particular de Loja, San Cayetano Alto s/n, Loja 1101608, Ecuador; 2 Departamento de Ciencias Biológicas y Agropecuarias, Facultad de Ciencias Exactas y Naturales, Universidad Técnica Particular de Loja, San Cayetano Alto s/n, Loja 1101608, Ecuador; 3 Laboratorio de Investigación en Parasitología Veterinaria, Universidad Nacional Experimental Francisco de Miranda, Coro 4101, Venezuela

**Keywords:** goat, *Neospora caninum*, *Toxoplasma gondii*, *Cryptosporidium* spp., *Eimeria* spp., prevalence, risk factors, apicomplexans, Ecuador

## Abstract

**Simple Summary:**

*Neospora caninum*, *Toxoplasma gondii*, *Cryptosporidium* and *Eimeria* species are parasites of phylum Apicomplexa, which includes several protozoa affecting animals and humans. In Ecuador, the maintenance of goat health is a matter of utmost importance because it affects the economic welfare of the breeders. *N. caninum* and *T. gondii* cause reproductive problems in goats, leading to abortions or weak offspring. Severe diarrhea in kids and delays in growth are due to the *Cryptosporidium* and *Eimeria* species. Moreover, *T. gondii* and *Cryptosporidium* are zoonotic parasites with serious consequences for human health. The aim of this work was to determine, by serological and parasitological tests, the prevalence of these parasites and the risk factors for goat populations in Southern Ecuador. On some farms, the prevalence of *N. caninum* and *T. gondii* reached more than 50%; up to 17% of the kids were positive for *Cryptosporidium* and 90% of the goats were positive for the *Eimeria* species. The analysis of risk factors revealed differences according to the parasite species. Considering the zoonotic significance of these results, control and prevention measures are essential and constitute a warning to veterinarians and governmental institutions.

**Abstract:**

*Neospora caninum*, *Toxoplasma gondii*, *Cryptosporidium* and *Eimeria* cause severe impacts on the productivity of goat herds. The objectives of the present study were to establish the prevalence of these apicomplexans in goat farms from Ecuador; to evaluate a rapid test for *Cryptosporidium* diagnosis and to identify the risk factors associated with the infections. A questionnaire was designed to obtain information from 24 goat farms from Zapotillo, Garza Real, Cazaderos, Limones and Paletillas parishes in Ecuador. Blood (*n* = 388) and feces (*n =* 391) samples were collected. Indirect ELISA and standard parasitological assays were carried out to evaluate the seroprevalence of *N. caninum* and *T. gondii* and to detect oocysts of *Cryptosporidium* and *Eimeria*. The overall prevalence values of *N. caninum* and *T. gondii* were 12.11% and 18.20%, *Cryptosporidium* spp. and *Eimeria* spp. oocysts were detected in 10.49% and 89.51% of the total samples. A low correlation value was found between the results obtained by Ziehl-Nielsen and the rapid test. The multinomial logistic regression analysis revealed that vitamin supplementation, age of diarrhea, frequency of deworming, pasture area, presence of artiodactyls, domestic fowl, administration of sulfas, age group, body condition, abortions, type of pastures and the presence of cattle were risk factors according to the parasite species.

## 1. Introduction

The phylum Apicomplexa constitutes a broad group of microorganisms that includes more than 6000 named species of single-celled, obligate intracellular protozoan organisms that all have a parasitic life cycle [1], distributed among a wide diversity of animals. Many of these parasites have significant clinical and economic relevance since they cause important human and veterinary diseases worldwide [2]. There are apicomplexans that originally developed through an oral–intestinal cycle and are commonly referred to as coccidia, one of the most important groups of animal parasites. Coccidians *in sensu stricto* (e.g., *Eimeria, Cryptosporidium, Cystoisospora*) are considered host-specific with a simple one-host life cycle; infection is limited to the intestines and usually to the enterocytes. The life cycle of a coccidian parasite of cats, *Toxoplasma gondii*, probably evolved from a fecal–oral cycle. Some apicomplexans also acquired other forms of transmission, e.g., fecal–oral cycle, carnivorism and transplacentally, adapting to several hosts. The discovery of oocysts in cat feces led to the recognition of several new taxa of economically important *Toxoplasma*-like parasites (e.g., *Hammondia, Neospora, Sarcocystis*) [3]. Unlike *Eimeria* and *Cryptosporidium*, the life cycles of *Neospora caninum* and *Toxoplasma gondii* involve, in addition to the sexual stage in canids or felids as definitive hosts, tissue stages with multiple intermediate hosts.

Domestic dogs and wild canids are the definitive hosts of *Neospora caninum*, and several species participate in the life cycle as intermediate hosts, including goats [4]. *Neospora caninum* is responsible for reproductive problems in ruminants; the disease is considered a major cause of abortion in cattle worldwide, but embryonic resorption, fetal mummification, fetal maceration, stillbirth and clinically healthy (but infected) kids may also occur [5]. The results of a systematic review of 22,234 goats from 18 countries showed a higher proportion of seropositive animals in the Americas compared to other regions [6]. Ecuador was not included in that review and, until the present study, there was no published data about goat neosporosis.

*Toxoplasma gondii* is a zoonotic parasite that is able to infect probably all warm-blooded animals and humans; one-third of the human population is chronically infected with the parasite [3]. Oocysts of *T. gondii* are formed only in cats, including both domestic and wild felids, but less than 50% of cats shed oocysts after ingesting tachyzoites or oocysts, whereas nearly all shed oocysts after ingesting cysts present in tissues (e.g., meat, milk) of the intermediate hosts [7]. Infection with the parasite is an important cause of neonatal mortality in small ruminants, resulting in reproductive and economic losses worldwide. Among small ruminants, goats appear to be more susceptible to clinical toxoplasmosis, and even adult goats have died from acute infection. Congenital toxoplasmosis in small ruminants can kill the fetus [7]. Reproductive failure is one of the important clinical consequences of *T. gondii* infection acquired during primary infection. Kids may be mummified, macerated, aborted, stillborn, or may be born weak or die soon after birth [3]. Sheep and goats are important sources of infection for humans due to their role as intermediate hosts; the consumption of infected milk, raw fresh cheese or meat can facilitate the zoonotic transmission; ingestion of undercooked infected lamb is recognized as risk factor for *T. gondii* infection in Europe and particularly in pregnant women [3]. In Guayaquil, Ecuador, a study of pregnant women receiving prenatal care revealed a prevalence of toxoplasmosis of up to 73%; consumption of undercooked meat was the main risk factor for acquiring the infection [8].

*Cryptosporidium* and *Eimeria* species are transmitted by accidental ingestion of highly resistant and environmentally stable oocysts present in food and water [9,10]. *Cryptosporidium parvum, C. hominis, C. ubiquitum* and *C. xiaoi* have been identified in goats; the common occurrence of zoonotic *C. parvum* and *C. ubiquitum* in small ruminants has raised public health concerns over cryptosporidiosis [11]. The impact of the disease depends on several factors, e.g., the susceptibility of the animals, the presence of carrier status and the stability of infection. The disease is difficult to control due to the reproductive ability of the parasite, the lack of vaccines, effective drugs and cumbersome diagnostic procedures [12]. In ruminant livestock, including goats, parasitic infection affects growth and production. The disease also exerts a substantial burden on the health and growth of children in developing countries [9]. Cryptosporidiosis is characterized by self-limiting diarrhea in immune competent individuals, but in immune compromised patients, the disease could be fatal.

The disease caused by the genus *Eimeria* is commonly known as coccidiosis. *Eimeria* species are found worldwide under different habitats and husbandry conditions and may be considered a highly relevant factor in intestinal disease of young animals [10]. Several species of *Eimeria* parasite cattle, small and wild ruminants, but there is no cross infection between species due to the strict host specificity [13]. Many factors influence the pathologic and clinical outcome of coccidiosis: mixed infections with several species, a short life cycle associated with an extraordinary reproductive capacity of the parasite, inflammatory immune responses, concomitant infections with other pathogens, management practices and stress [10]. Depending on the type of management, coccidiosis might affect 100% of goat kids of 4–10 weeks, disturbing animal health and the productivity of farms. *Eimeria*-infected goat kids show intestinal symptoms, from non-hemorrhagic to severe hemorrhagic diarrhea, dehydration, weight loss and growth delay, particularly during the weaning period [14].

In the countries where the majority of goats are found, most farmers are of lower socioeconomic status; locally adapted goat breeds are raised for milk and meat, and in dry and drought-prone areas, goat milk is often the only protein source in children’s diets [15]. In Southern Ecuador, the province of Loja is characterized by a pronounced dry season and limited natural resources [16]. In Zapotillo Canton, most of the livestock activity corresponds to goat breeding: 28,000 goats are distributed in parishes named Limones (50.22%), Cazaderos (15.94%), Zapotillo (14.21%), Garza Real (10.97%), Paletillas (4.47%) and Bolaspamba (4.17%) [17]. Farms are characterized by extensive management with animals of low genetic quality; the main activity is the production and commercialization of milk. Goat breeding is managed under the traditional extensive system; occasionally, animals receive corn husks, corn cobs, carob beans and crop residues as a supplementary diet [18].

Due to the impact of these parasitic diseases on goat health, the economic performance of farms and the human population that depends on goat breeding, the present work aimed to estimate the seroprevalence of *Neospora caninum* and *Toxoplasma gondii* by ELISA tests and the prevalence of *Cryptosporidium* spp. and *Eimeria* spp. by parasitological techniques; to evaluate a rapid immunochromatographic test for the diagnosis of *Cryptosporidium* and to identify the risk factors associated with infections in goat herds from Ecuador.

## 2. Materials and Methods

### 2.1. Study Area

**Figure 1:**
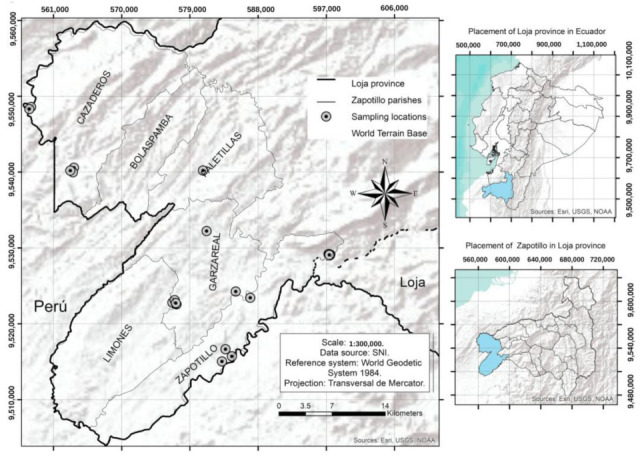
Sampling locations in parishes from Zapotillo Canton, Ecuador.

The research was carried out on 24 goat farms from Zapotillo, Garza Real, Cazaderos, Limones and Paletillas, parishes of Zapotillo Canton, Loja Province, Southern Ecuador (Figure 1). The farms were selected by the RAND function in Excel^®^ (v. 17.0, Microsoft, Redmond, WA, USA) using the database provided by the Agricultural Public Information System (SIPA, by the acronym in Spanish) of the Ministry of Agriculture and Livestock of Ecuador [19]. Local breeders used to release the goats every day to forage freely in the forest, and at sunset, the animals returned to the dirt-floored stables located near the owner’s houses [20].

### 2.2. Sampling

A total of 388 blood samples for serological diagnosis of *N. caninum* and *T. gondii* were collected from the jugular vein using the BD-Vacutainer^®^ (Becton Dickinson, Mississauga, ON, Canada) system in tubes without anticoagulant. Sixteen goats aged over six months were sampled on each farm. After centrifugation of the blood samples, sera were collected and stored in aliquots at −20 °C until use.

A total of 391 fecal samples were collected directly from the rectum using clean plastic bags. A minimum of sixteen samples were taken per farm: eight samples from kids under six months old and eight samples from adults over six months old [21]. Some farmers accepted the sampling of blood but did not accept the sampling of feces from their animals, so the minimum sample number was completed on other farms. The fecal samples to be used in the detection of *Eimeria* spp. and *Cryptosporidium* spp. by flotation and McMaster techniques were preserved in 10% formalin. Fecal samples to be used in the *Cryptosporidium* rapid test were stored without any additives at −20 °C until use, according to the manufacturer’s instructions. A FAMACHA card was used to assess the level of anemia in goats by measuring the color of the ocular mucosa [22].

Data concerning each animal were registered on a separate sheet; each of the blood and fecal samples were numbered and registered along with the animal’s age (verified by dentition), sex, FAMACHA score and any comments about the animal.

### 2.3. Questionnaire

An organized questionnaire was designed to collect general information about the farms (area, pastures, soil irrigation, number, sex and age of goats), management procedures (presence of other species, drinking and feeding troughs, cleaning of pens, mineral/vitamins or food supplementation, goat milk and meat consumption), disposal of abortion products) and animals (sanitary management, abortions, symptoms such as diarrhea, emaciation or anorexia). All of the questionnaires were completed face-to-face by the same interviewer. Participation was voluntary.

### 2.4. Serological Assays

Detection of Antibodies Anti-*Neospora caninum* and *Toxoplasma gondii* by ELISA

Serological analysis of goat sera was performed using indirect ELISA kits for the detection of goat IgG antibodies against *N. caninum* and *T. gondii* (IDEXX^®^ NeosporaAb and IDEXX^®^ ToxotestAb, Westbrook, ME, USA, respectively). A total of 388 goat blood sera were tested in *N. caninum* ELISA and 368 in *T. gondii* ELISA, excluding females which had not reached the minimum reproductive age of six months and males.

To detect antibodies against *N. caninum*, 90 μL of sample diluent and 10 µL of undiluted sample sera, positive and negative control sera were dispensed into appropriate wells of the plates precoated with antigen. Plates were incubated at 37 °C for 60 min. Following incubation, three washes with 300 μL per well were carried out using an ELISA plate washer (BioTek^®^ 50 TS8, Friedrichshall, Germany). After washing, 100 μL of conjugate was dispensed in each well and incubated at 37 °C for 60 min. The wells were washed again as described, and 100 μL of TMB-substrate was added; plates were incubated at 18–26 °C for 15 min. The reaction was stopped with 100 μL per well of stopping solution. Plates were read at 450 nm in an ELISA reader (BioTEk^®^ ELx800, Friedrichshall, Germany) using Gen5^®^ software (BioTek^®^, Friedrichshall, Germany). Positive and negative control sera were tested in duplicate according to the commercial test.

For detection of antibodies against *T. gondii*, 100 μL of pre-diluted (1:400) sera samples and controls were dispensed into wells of the plate precoated with antigen. The rest of the procedure was conducted as described above for *N. caninum*.

Interpretation of results was made using the formula:

Sample/Positive (S/P) % = 100 × ((sample OD value at 450 nm − X OD negative control)/ (X OD positive control − X OD negative control))(1)

A sample was considered negative to *N. caninum* if S/P % < 30; suspect, 30 ≤ S/P % < 40 or positive, if S/P % ≥ 40.

A sample was considered negative to *T. gondii* if S/P % < 20; suspect 20 ≤ S/P % < 30; weak positive 30 ≤ S/P % < 100 or positive if S/P % ≥ 100.

### 2.5. Parasitological Tests

#### 2.5.1. Flotation Technique

The sucrose flotation method [23] was used to detect oocysts of the genus *Eimeria.* Briefly, two grams of feces were mixed thoroughly with 30 mL of Sheater´s flotation solution (density 1.27) in a cup and strained through two layers of gauze; the filtered solution was placed into a test tube to completely fill it, ensuring that a slightly convex meniscus was formed. A 22 × 22 mm coverslip was placed on the tube, left for 10 min, removed and placed on a glass slide. The entire coverslip was examined under a light microscope at 400x magnification.

#### 2.5.2. McMaster Technique for *Eimeria* Oocyst Counting

The filtered solution of feces obtained in the flotation technique was also used to be transferred into the McMaster chamber for oocyst counting [23]. The quantitative results were recorded as oocysts per gram of fecal (opg) value. The level of infection was estimated as a Bangoura and Daugschies score [24]: 0, no oocysts detectable; 1, ≤100 opg; 2, ≤1000 opg; 3, ≤10,000 opg; 4, ≥10,000 opg.

#### 2.5.3. Identification of *Cryptosporidium* spp. in Fecal Samples

The formalin-ethyl acetate concentration method (FEA) modified by Weber et al. [25] was used to confirm the presence of *Cryptosporidium* in fecal samples. Briefly, four milliliters of the formalin-fixed feces suspension, 6 mL of 10% formalin and 3 mL of ethyl acetate were placed into a 15-mL centrifuge tube, shook thoroughly and centrifuged at 500× *g* for 5 min. The supernatant was decanted and sediment was suspended in 5 mL of deionized water, layered carefully over 5 mL of saturated sodium chloride and centrifuged at 500× *g* for 10 min. Approximately 3 mL of the top layer was removed and discarded. The remainder of the top layer and approximately 0.5 mL of the medium layer containing saturated sodium chloride were removed and washed in 13 mL of deionized water by centrifugation at 500× *g* for 10 min. The smears were made from the sediment and stained by the modified Ziehl-Neelsen (ZN) procedure for *Cryptosporidium* [26]. Slides were dried out at room temperature and fixed with methanol for 3 min, washed with distilled water and covered with carbofuchsin for 10 min; after washing with tap water, acid alcohol was placed on the slides for 30 s, washed again and counterstained with 1% methylene blue for 1 min. After washing, the slides were allowed to dry at room temperature and checked under the microscope at 1000× magnification. A fecal sample of a previously tested-positive animal was used as a control.

### 2.6. Detection of Cryptosporidium spp. in Fecal Samples by a Rapid Test

The RIDA^®^QUICK *Cryptosporidium* test (R-Biopharm AG, Darmstadt, Germany) was used to evaluate the feasibility of *Cryptosporidium* diagnosis in the field. Fecal pools from each farm from two groups of age (animals under six months and over six months old) were used. Fifty milligrams of pooled, thawed out feces, manually homogenized, were suspended in buffer (included in the kit) and allowed to settle down for three minutes until a clear supernatant was formed; 200 μL of supernatant was placed in the inlet hole of the cassette. Reading was carried out five minutes later; the control line and the line corresponding to a positive result were verified and recorded. The results were compared with those obtained by ZN.

### 2.7. Data Analyses

The SPSS^®^ IBM^®^ package for Windows (version 24; SPSS, Chicago, IL, USA) was used for statistical analyses. The results of serological and parasitological tests were included in a data sheet (Excel^®^ v. 17.0, Microsoft, Redmond, WA, USA) along with the variables obtained through the questionnaire (Table A1). The SI function was applied to assign a value of one if a positive number different from zero was found in the cell of the previous column. For descriptive analyses, parish and farm level prevalence were calculated. In addition, non-parametric tests were carried out because seroprevalence of *N. caninum* and *T. gondii* and prevalence of *Cryptosporidium* spp. and *Eimeria* spp. did not follow a normal distribution (Kolmogorov–Smirnov Z = 0.205, *p* < 0.05; 0.529, *p* < 0.05; 0.523, *p* < 0.05, respectively).

To establish differences between variables of two groups, e.g., animals <6 months and >6 months, a non-parametric Mann–Whitney U test was used. A non-parametric ANOVA (Kruskal–Wallis) was used to establish differences between variables with more than two groups, e.g., parishes. To assess the significance of each variable related to positivity, a Kruskal–Wallis test was used [21] with a 95% confidence interval and a *p*-value < 0.05 [27].

Nominal variables were coded numerically, assigning a number to each category with the mean as the cut-off point. A binary value was assigned to the dichotomous variables (1 if the answer was yes, and 0 otherwise). Regarding numerical variables, central tendencies and dispersion statistics were calculated. Variables with more than two categories were transformed into dichotomous variables using a reference category. In the cases of *N. caninum* and *T. gondii*, the serum which resulted as “suspect” or “weak positive” by ELISA was considered as positive due to the impossibility of obtaining duplicated samples. Consequently, the dichotomy of variables was maintained in the statistical analysis.

To determine risk factors, 49 variables (Table S1) related to management and characteristics of the farm and animals were paired to the prevalence of each of the parasites. Some variables were excluded at the time of analysis (those related to the farm or dichotomous variables with a frequency value higher than 95%).

A selection of variables was carried out to accomplish the multivariable model. For categorical variables, a Chi-square test was used, selecting variables with *p* < 0.2. For numerical variables, a Kolmogorov–Smirnov test was used, selecting variables with a *p*-value < 0.05. The variables selected by univariable analysis were subjected to a multinomial logistic regression model [28]. The logistic regression analysis was carried out using non-automatic variable selection and stepwise entry to determine the significance (*p* < 0.05) of each variable. Results were presented as odds ratios (OR) adjusted to a 95% confidence interval (CI).

## 3. Results

### 3.1. Seroprevalence of Neospora caninum and Toxoplasma gondii

The overall seroprevalence of *N. caninum* was 12.11% (47/388) in 10 of the 24 farms. Seroprevalence values per farm ranged from no seropositive animals in 14/24 of the farms to 81.25% of seropositive animals in a single farm from Cazaderos parish (Table A1).

The seroprevalence value of *T. gondii* was 18.20%; values ranged between 11.11 (Cazaderos parish) and 23.03% (Zapotillo parish) (Table 1) (*p* > 0.05) in 20/24 farms. The maximum value of *T. gondii* seroprevalence per farm was 61.11% (Table A1). No significant differences were found between sex of the animals or breed phenotype (*p* > 0.05).

### 3.2. Prevalence of Cryptosporidium spp. and Eimeria spp.

*Cryptosporidium* spp. oocysts were detected in 10.49% (41/391) of the samples and in 91% (20/22) of the farms. The highest prevalences of *Cryptosporidium* spp. were found in Garza Real (12.96%) and Zapotillo (10.70%); prevalence values per farm ranged from 3.85% to 35.29% (Table 1). Oocysts were observed in 16.88% (27/160) of kids <6 months and 6.06% (14/231) of goats >6 months.

*Eimeria* oocysts were observed in 89.51% (350/391) of the samples; 85.71% (198/231) in adult animals and 95% (152/160) in kids; within the farms, the prevalence ranged from 58.82% to 100%. The highest prevalence was observed in Paletillas (100%, 17/17) and Garza Real (94.43%, 102/108) and the lowest in Limones (80%; 12/15) (Table 1). Significant differences were found between parishes, farms (*p* < 0.05), adults (>6 months) and kids (<6 months) (Z = −2.943, *p* < 0.05), with a higher frequency of positivity in kids (*p* < 0.01). There were no significant differences between males and females or animal phenotype (*p* > 0.05).

The average oocyst count was 33.54 ± 100.9. Of the 391 feces samples, 41 showed no oocysts and were classified as score 0; 325 samples were classified as score 1 (≤100 opg), 24 samples as score 2 (≤1000 opg) and 1 sample with 1012 opg as score 3 (≤10,000 opg).

### 3.3. Prevalence of Cryptosporidium spp. Using the Rapid Test

The rapid tests were performed on pools of feces from two age groups on each farm. Of the 22 farms tested, seven were positive to the rapid test (3 < 6 months; 4 > 6 months). No correlation was found between the results obtained by Ziehl-Nielsen and those obtained by the rapid test in samples from kids (r = −0.043, *p* > 0.05). There was a weak but non-significant correlation between the prevalence of *Cryptosporidium* spp. in adults and the rapid test (r = 0.327, *p* > 0.05). Significant differences were found between results obtained by both Ziehl-Nielsen and rapid tests on the same farms (*p* < 0.05).

### 3.4. Mixed Infections

Two hundred and forty-four animals were infected with two of the four apicomplexans; 30 animals were infected with three parasites and six with all four species (Table 2). Regarding infections with two species, the highest prevalence value was 38.62% (151 animals from 22 farms with both *Cryptosporidium* spp. and *Eimeria* spp.); the lowest was 2.72% with *T. gondii* and *N. caninum* (10 animals from 6 farms). *T. gondii, Cryptosporidium* spp. and *Eimeria* spp. were observed in 4.62% of the animals from 10 farms; lower prevalences were observed with *N. caninum, T. gondii* and *Eimeria* spp. (1.90%) or *N. caninum, T. gondii* and *Cryptosporidium* spp. (1.63%) (Table 2).

## 4. Analysis of Risk Factors

### 4.1. Risk Factors for Neospora caninum and Toxoplasma gondii Infections

The frequencies of 49 categorical variables are presented in Table S1. Univariable analysis revealed 26 variables associated with farm management and six related to animals (e.g., body condition, ectoparasites, abortions, milk production) as risk factors for *N. caninum* seropositivity (Table A1 by a Chi-square test). Ten numerical variables (*p* <0.05) were selected by a Kolmogorov–Smirnov analysis: animal age, farm area, pasture area, number of animals (males, females and kids), mortality/year (adults, kids) and milk production.

Twenty-two variables were considered as risk factors for *T. gondii* seropositivity by univariable analysis: 19 related to farm characteristics and managment and three to animals (age of animals with diarrhea and duration of diarrhea, infection with louses) (Table A1); from these, 16 variables are common for both *N. caninum/T. gondii*, three of them related to goats (age of animals with diarrhea and duration, infection with louses). By a Kolmogorov–Smirnov analysis, nine variables (*p* < 0.05) were selected: animal age (months), farm area, pasture area, number of animals (males, females), abortions, total area of the farm, partial area, mortality/year (adults, kids) and mortality of adult goats.

The multivariable logistic regression analysis suggested four variables as risk factors for *N. caninum* infection (vitamin supplementation, age of diarrhea, frequency of deworming and known pasture area) (Table 3); for *T. gondii* infections: presence of artyodactils, presence of domestic fowl in the farms and administration of sulfas (Table 3).

### 4.2. Risk Factors for Cryptosporidium spp. and Eimeria spp.

Ten variables were revealed as risk factors for *Cryptosporidium* by univariable analysis: age group, body condition, dairy farm, irrigation, presence of cats, facilities, ventilation, infection with louses, abortion products in pastures and technical visits; animal age (*p* < 0.05) was selected by Kolmogorov–Smirnov analysis. Nineteen categorical variables were selected for *Eimeria* infections: age group, FAMACHA results, presence of cattle, dairy farms, irrigation, presence of cats, frequency of cleaning, water troughs, plastic troughs, infection with louses, food supplementation, tire troughs, administration of sulfas, abortion products in pastures, milk production values, abortions (%), type of pasture, fertilization and dogs’ consumption of abortion products (Table A3); animal age and pasture area (*p* < 0.05) were selected by Kolmogorov–Smirnov analysis to be further considered as risk factors.

The multivariable logistic regression analysis suggested two variables as risk factors for *Cryptosporidium* spp. infection (age group and body condition) (Table 4); for infections with *Eimeria* spp., percentage of abortions/total females, type of pastures and presence of cattle (Table 4).

### 4.3. Risk Factors for Mixed Infection

Three variables related to general and sanitary management of the farms were found as risk factors for mixed infections: intensive management, frequency of cleaning >3 months and administration of sulfas (Table 5). No statistical differences were found between the other variables and infections with three or four parasites.

## 5. Discussion

This work reported the prevalence of four apicomplexan parasites that affect goats in farms in Ecuador; the risk factors associated with the infections are also presented. They all have negative consequences on the farms’ economy, either due to the effect on weight gain, secondary infections and mortality because of the intestinal damage by *Cryptosporidium* and *Eimeria* species or to abortions and weak offspring by *Neospora caninum* and *Toxoplasma gondii* [5,7,10,12]. Furthermore, as zoonotic parasites (some species of *Cryptosporidium, T. gondii* and probably *N. caninum* [29]), they pose a serious concern to human populations.

It is well-known that bovine neosporosis is an abortigenic parasitic disease that causes severe economic losses on cattle farms. However, the economic and epizootiologic importance of *Neospora caninum* infection in goats remains in doubt as the pathogenesis of caprine neosporosis is mostly unknown [30]. Until the present work, there have been no reports of neosporosis in goats from Ecuador; 21–23% of seroprevalence values were shown in cattle herds from Santo Domingo, Cañar and Azuay provinces [31,32]. The seroprevalence value of *N. caninum* found in this work (12.11%) was higher than those reported in Italy (5.7%), China (8.55%) or in Romania (2.3%) [33,34,35]. In Brazil, 26.11% of seroprevalence was described [36]. It is remarkable that two farms in the present study showed more than 50% seropositivity to *N. caninum.*

In the present study, vitamin supplementation and regular deworming were determined as risk factors. A common management practice is to place supplements (dried feeds, vitamins or minerals) in a container and replace it when depleted; deworming products or dried supplements are also kept in a warehouse where dogs and domestic fowl used to sleep. In most goat farms in Ecuador, no aseptic measures are taken in drug administration by farmers; bottles containing medicines are found in pens or bins; thus, oocysts present in dried feces can be carried by the wind, insects or birds and can be attached to them [37,38]. Diarrhea episodes 30 days after birth and a known grazing area were also found as risk factors. In cattle, co-infection of *N. caninum* with other pathologies, such as herpesvirus BHV-1 and stress-causing immunosuppression, has been reported [33,39]. The animals that are not allowed to graze freely or with a grazing area delimited by physical barriers are more exposed to oocyst ingestion [40].

Due to the high frequency of domestic dogs (95.8%), the odds ratio could not be determined. Canids are the final hosts of *N. caninum*, so their presence would be expected to be a risk factor for goats [30,41]. Thus, this variable should not be ruled out as a risk factor for *N. caninum*.

There are a large number of papers regarding *Toxoplasma gondii* seroprevalence in goats using techniques such as ELISA, immunofluorescence or microaglutination tests; despite several common antigens, there are no cross-reactions between *N. caninum* and *T. gondii* [40]. The findings of *T. gondii* seroprevalence around the world in the period 2009–2020 have been excellently summarized by Dubey [3]. Seroprevalence values ranged between 1.5 and 73.8% worldwide (including those obtained in the present work), but it is difficult to compare the findings due to differences, e.g., in diagnostic methods, farm and animal management and the presence of domestic and wild felids. A real zoonotic risk was found in two goat farms sampled in this investigation, with seroprevalence values of 61.11% and 50%. Recently, a systematic review of the literature and a meta-analysis regarding the seroprevalence of *T. gondii* in 55,317 goats from 75 reports did not include data from Ecuador, which emphasizes the importance of the present study [42].

The only known definitive hosts for *Toxoplasma gondii* are members of the family Felidae. Domestic cats are the major source of environmental contamination with oocysts; however, it is a difficult matter to prevent cats from living or loitering on farms that have grazing stock [7]; farmers also have cats at farms to avoid rats. In our study, the presence of domestic fowl and artiodactyls and administration of sulfa drugs were found to be risk factors. The presence of domestic fowl is a risk factor due to their consumption habits and the possibility of being vectors of oocysts from cat feces, similar to what occurs with the dispersion of *Neospora* oocysts from dog feces. It has been shown that domestic chickens can host *T. gondii* [43] and so contribute to the cycle in a predator–prey relationship or by consumption of raw chicken meat provided to the cat by the farmer. A similar reason for poor sanitary management through treatment administration as a risk factor in *N. caninum* applies to the administration of sulfa drugs for *T. gondii* [37] Sporulated oocysts survive for long periods under moderate environmental conditions and can be spread by the erosion of topsoil and mechanically by flies, cockroaches, dung beetles and earthworms [7]. Otherwise, sulfonamides, such as sulfadiazine, were found to have important inhibitory effects on *T. gondii* [44]; however, it should not be discarded that an induced resistance to sulfas was previously described [45]. In Ecuador, the resistance to drugs is a concern for veterinarians; veterinary drugs are sold over-the-counter without supervision.

The presence of artiodactyls as a risk factor for *Toxoplasma gondii* is an interesting topic of discussion, and no information is available from Ecuador. Aston et al. [46] evaluated *T. gondii* seropositivity in artiodactyls hunted in Peru and found antibodies in peccaries *Pecari tajacu, Tayassu pecari*, brocket deer species *Mazama americana*, *Mazama gouazoubira* and *Tapirus terrestris* (lowland tapir), which probably served as reservoirs in their natural habits. Farmers used to consume some wild species as bush meat and eventually feed the domestic cats with the remains, thus maintaining the cycle on the farms. The main wild ruminant species, hunted and destined for human consumption in Spain (red deer and roe deer), showed high antibody titers against *T. gondii*, suggesting that undercooked game meat should not be consumed by humans or be used to feed cats [47]. Further studies are necessary to elucidate the *T. gondii* infection levels in meat and derived products from wild artiodactyl species in Ecuador.

Cryptosporidiosis is one of the major health problems in neonatal goats kids because it causes retarded growth, decreased feed efficiency, delayed maturity, loss of fertility and mortality levels could reach up to 40% [48]. The presence of *Cryptosporidium* species is also a risk for the human population because there are species with zoonotic potential [15]. Several works reported higher prevalence values in kids compared to adult goats [49,50,51,52]. The prevalence of *Cryptosporidium* spp. found in this work ranged between 5.26 to 20% (there were no positive samples in 2/22 farms), indicating that there is a widespread dissemination of *Cryptosporidium* in goat farms from Zapotillo Canton.

In the present study, a rapid commercial assay was used to detect *Cryptosporidium* without requiring analysis in a laboratory. A low coincidence was found between the ZN technique and rapid tests in both kids’ and adults’ samples. The RIDA^®^QUICK *Cryptosporidium* test identifies an antigen of *Cryptosporidium parvum*; however, there may be other species of *Cryptosporidium* parasitizing goats. In Spain, a report showed that 71.4% of the samples were positive for species other than *C. parvum*: *C. xiaoi* and *C. ubiquitum* [47]. In the United Kingdom, *C. hominis* has been reported in goats with a potential risk for people in contact with infected animals [53]. Other reasons may justify the low concordance between both assays. According to the manufacturer, a negative result of the immunochromatographic test does not exclude a possible infection with *C. parvum*, which may be due to a low amount of antigen in the sample. In Spain, two commercial immunochromatographic methods were reported to have significant differences in sensitivity and specificity [54]. Other studies suggested that immunochromatographic assays used for detection of *Cryptosporidium* in feces were specific to a certain antigen and did not provide information on other species or genotypes involved [55,56], for which molecular techniques should be performed [15].

Two variables were found as risk factors for *Cryptosporidium* spp.: age group (kids under 6 months old) and a “regular” body condition of the animals. The main source of infection for newborn kids is contact with feces from adult animals with subclinical infections [57], especially in females during the peripartum period, when oocysts are most frequently shed. A “fair” body condition (<3) was determined as a risk factor for *Cryptosporidium*: in one month, the difference in weight gain of an infected animal compared to a healthy one can be up to 1.48 kg [11].

In this study, each farm in each parish was positive for *Eimeria* spp. as expected and reported worldwide. The genus *Eimeria* is distributed globally, and the infection rates can reach more than 90% in some areas. The prevalence is largely dependent on farm management because oocysts require moist and dark environments to sporulate, which is a typical situation in goat pens in Ecuador. Under conditions that promote *Eimeria* development, the accompanying clinical symptoms include low feed conversion rate, weight loss and lethargy [58].

In this research, most of the samples were ranked at score 1 (less than 100 opg), meaning that goats were in subclinical infection. The economic impact of coccidiosis in small ruminants is not well documented; in tropical regions with extensive breeding zones and animals of local origin, subclinical coccidiosis with poor weight gain as a symptom is probably not of major importance compared to other infections [14]. The coprological examinations should be quantitative and allow the diagnosis of the most pathogenic species of *Eimeria* found in feces. Such identification is done on the basis of the morphology of the sporulated oocysts and their structures and estimation of sporulation periods [10]. However, despite the general relationship between clinical coccidiosis and high excretion of oocysts, a cut off for oocysts values is difficult to calculate because of the differences in pathogenicity of each of the nine *Eimeria* species identified in goats [14]. Certainly, the identification of the *Eimeria* species would have offered invaluable information about *Eimeria* goat species from Ecuador, but this goal could not be achieved. It is important to highlight that coccidial infection causes an imbalance in the intestinal microbiota, which not only leads to a decrease in food intake and reduced absorption but also increases the susceptibility of the organism to secondary infections and impairs the intestinal mucosal barrier function [59]. Control measures for coccidiosis should be taken at farm level no matter which species are involved.

Four variables were identified as risk factors for *Eimeria* in parishes from canton Zapotillo: kids younger than 6 months, farms with abortion problems, the presence of cultivated pastures and the absence of cattle. Coccidiosis is a disease that primarily affects young animals in all species, and goats are not an exception [10,60,61]. Abortions were identified as a risk factor because the concomitant immunity developed by ruminants against *Eimeria* spp. is impaired and the peak of excretion occurs in the peripartum period [62]. A large number of *Eimeria* oocysts released in feces by immune compromised animals act as a source of contamination for other members of the herd. The use of cultivated grassland is related to a medium-high level of breeding “intensification” which is considered a risk factor for the presence of *Eimeria* spp. [14]. In Ecuadorian goat farms, corn husks and cobs from domestic crops are also placed on the ground or in the feeders as a supplement, increasing the risk of infection.

The sampling was carried out during the transition from the dry to the rainy season and some tree and shrub species were releasing their seeds to the ground. Goats living in arid or semi-arid areas with seasonal rainfall encounter abundant leaf supplies in the rainy season, but they are scarce in the dry season, being forced to graze at ground level in search of small plants and tree seeds [63]. If cattle are not present, there is no competition for food, and goats are more prone to ingesting oocysts that are at ground level [40].

In this study, mixed infections were also considered. Few studies show co-infections by protozoan in goats, despite the consequences they may have in the productivity of farms. For example, co-infections with *Neospora caninum* and *Toxoplasma gondii* have been scarcely reported. Hassig et al. [64] detected reproductive problems in a flock of sheep that had been evaluated independently for *T. gondii* or *N. caninum* and subsequently turned out to be mixed infections. In buffaloes, infection with *N. caninum* is associated with abortion and the presence of retained fetal membranes. Infection with *T. gondii* has been associated with an increase in days open; in the case of co-infections with both pathogens, the effects on the animals could be related to both abortion and embryonic death [65]. In this study, mixed infections for both species were found in 2.72% of the goats, but when assessing infections with three or four of the parasites, it was found that both *T. gondii* and *N. caninum* co-infected a greater number of animals (7.88%). In the state of Paraná, Brazil, the frequency of antibodies to both species was 3.0% (19/629) from 32 goat farms [66].

A small number of reports included estimations of co-infections by *Cryptosporidium* spp. and *Eimeria* spp. in goats. The prevalence of both parasites is age-related, but simultaneous infection seems not to be a very common scenario [67]. However, in this study, 150 samples/391 had mixed infection with *Eimeria* and *Cryptosporidium*, which represents an important number to consider in control measures.

To the best of the authors’ knowledge, there are no reports of co-infections with the four apicomplexans in goats as carried out in this study. All the risk factors are related to sanitation and farm management. The four apicomplexans have an oral route of infection: restricted grazing/bedding areas, poor cleaning of pens and keeping treatment bottles in inadequate places increase the exposure of animals to infective oocysts.

Finally, it would be interesting to assess whether host competition (host resources, host immune responses or interference between species) exists in these mixed infections. This is a topic that remains open for further research.

## 6. Conclusions

In this work, the prevalence of four parasites of phylum Apicomplexa, *Neospora caninum, Toxoplasma gondii*, *Cryptosporidium* spp. and *Eimeria* spp., and the risk factors associated with infection in goat farms in Ecuador are reported. The results showed high prevalence values in some farms, and in the case of zoonotic *T. gondii*, the results revealed a severe risk for human consumers of milk and undercooked meat from goats. Low association values were found between the Zielh Neelsen assay and the rapid test used to detect *Cryptosporidium* in both kids’ and adults’ samples. Risk factors for these parasitic diseases include several variables related to farm management and individual characteristics such as age of animals, presence of diarrhea or abortions. These results suggest the importance of control measures, epidemiological surveillance and education of farmers with the participation of governmental entities and private veterinarians to reduce the economic losses in goat farms and to maintain public health.

Based on the authors’ knowledge, this is the first report of *Neospora caninum, Toxoplasma gondii* and *Cryptosporidium* spp. and mixed infections in goat farms from Ecuador.

## Figures and Tables

**Table 1:** Seroprevalence of *Neospora caninum* and *Toxoplasma gondii* and prevalence of *Cryptosporidium* spp. and *Eimeria* spp. in goats from parishes of Zapotillo Canton, Ecuador.

Parish	Seroprevalence		Prevalence	
	*N. caninum* ^1,2^	*T. gondii* ^1,2^	*Cryptosporidium* spp. ^1,2^	*Eimeria* spp. ^1,2^
Garza Real	17.78 (16/90)	16.67 (15/90)	12.96 (14/108)	94.43 (102/108)
Zapotillo	4.79 (8/167)	23.03 (38/165)	10.70 (20/187)	85.03 (159/187)
Limones	2.78 (1/36)	14.81 (4/27)	0.00 (0/15)	80.0 (12/15)
Paletillas	0 (0/14)	14.29 (2/14)	5.88 (1/17)	100.0 (17/17)
Cazaderos	27.16 (22/81)	11.11 (8/72)	9.38 (6/64)	93.75 (60/64)
Total	12.11 (47/388)	18.20 (67/368)	10.49 (41/391)	89.51(350/391)

^1^ Values presented as percentages. ^2^ Inside the brackets, number of positive goats/total of goats sampled in the parish.

**Table 2:** Prevalence of mixed infections with *Neospora caninum, Toxoplasma gondii, Cryptosporidium* spp. and *Eimeria* spp. in sampled goats from canton Zapotillo, Ecuador.

Mixed Infections	Prevalence (%)	Animals/Farms
*Cryptosporidium* spp. and *Eimeria* spp.	38.62	151/22
*T. gondii* and *Eimeria* spp.	7.88	29/13
*N. caninum* and *Eimeria* spp.	5.93	23/8
*T. gondii* and *Cryptosporidium* spp.	4.35	16/9
*N. caninum* and *Cryptosporidium* spp.	3.87	15/8
*T. gondii* and *N. caninum*	2.72	10/6
*T. gondii, Cryptosporidium* spp. and *Eimeria* spp.	4.62	17/10
*N. caninum, T. gondii* and *Eimeria* spp.	1.90	7/5
*N. caninum, T. gondii* and *Cryptosporidium* spp.	1.63	6/5
*N. caninum, T.gondii, Cryptosporidium* spp. and *Eimeria* spp.	1.63	6/5

**Table 3:** Risk factors associated with *Neospora caninum* and *Toxoplasma gondii* in goats from Ecuador obtained by multivariable logistic regression analysis.

Variable	Category	*N. caninum*			*T. gondii*		
		*p* ^a^	OR ^b^	CI ^c^ _95%_	*p* ^a^	OR ^b^	CI ^c^ _95%_
Supplementation with vitamins	Yes	0.001	40.96	2.4–700.6	--	--	--
	No	*	*	*	--	--	--
Age of diarrhoea	>30 days	<0.0001	11.83	2.9–46.8	--	--	--
	<30 days	*	*	*	--	--	--
Frequency of deworming	Regular	0.001	42.31	4.6–392.8	--	--	--
	Irregular	*	*	*	--	--	--
Known pasture area	Yes	0.021	25.16	1.6–390.4	--	--	--
	No	*	*	*	--	--	--
Presence of artiodactyls	Yes	--	--	--	0.001	2.943	1.5–5.7
	No	--	--	--	*	*	*
Domestic fowl	Yes	--	--	--	0.009	2.428	1.3–4.7
	No	--	--	--	*	*	*
Administration of sulfas	Yes	--	--	--	<0.001	4.608	2.4–8.8
	No	--	--	--	*	*	*

^a^*p* value-Wald test; ^b^ Odds ratio; ^c^ Confidence interval; * Reference category.

**Table 4:** Risk factors associated with *Cryptosporidium* spp. and *Eimeria* spp. in goats from Ecuador, obtained by multivariable logistic regression analysis.

Variable	Category	*Cryptosporidium* spp.			*Eimeria* spp.		
		*p* ^a^	OR ^b^	CI ^c^ _95%_	*p* ^a^	OR^b^	CI ^c^ _95%_
Age group	<6 m	0.001	3.06	1.5–6.1	0.013	2.87	1.3–6.6
	>6 m	*	*	*	*	*	*
Body condition	Regular	0.016	2.48	1.2–5.2	--	--	--
	Good	*	*	*	--	--	--
% of abortions/total females	<10%	--	--	--	<0.0001	6.98	2.9–16.6
	>10%	--	--	--	*	*	*
Type of pasture	Cultivated	--	--	--	0.039	8.76	1.1–68.4
	Natural	--	--	--	*	*	*
Presence of cattle	No	--	--	--	0.002	3.98	1.7–9.4
	Yes	--	--	--	*	*	*

^a^ *p* value-Wald test; ^b^ Odds ratio; ^c^ Confidence interval; * Reference category.

**Table 5:** Risk factors of mixed infections with *Neospora caninum, Toxoplasma gondii, Cryptosporidium* spp. and *Eimeria* spp. in sampled goats from Zapotillo Canton, Ecuador.

Mixed Infections	Variable	Category	*p* ^a^	OR ^b^	CI ^c^_95%_
*N. caninum* and *Eimeria* spp.	Management	Intensive Extensive	0.005 *	3.546 *	0.12–0.68 *
*N. caninum* and *Cryptosporidium* spp.	Management	Intensive Extensive	0.013 *	3.891 *	0.09–0.75 *
*T. gondii* and *Cryptosporidium* spp.	Administration of sulfas	Yes No	0.003 *	4.713 *	1.69–13.13 *
*T. gondii* and *Eimeria* spp.	Frequency of cleaning	>3 months <3 months	0.015 *	5.912 *	1.20–5.54 *
*Cryptosporidium* spp. and *Eimeria* spp.	Management	Intensive Extensive	0.042 *	1.845 *	0.30–0.98 *
	Frequency of cleaning	<3 months >3 months	0.037 *	1.901 *	0.29–0.96 *
*T. gondii. Cryptosporidium* spp. and *Eimeria* spp.	Administration of sulfas	Yes No	0.010 *	4.375 *	1.42–13.51 *

^a^*p* value-Wald test; ^b^ Odds ratio; ^c^ Confidence interval; * Reference category.

## Data Availability

The data is available upon request to corresponding authors.

## Supplementary Materials

The following supporting information can be downloaded at: https://www.mdpi.com/article/10.3390/ani12172224/s1. Table S1. Frequency of categorical variables recorded in coprological and serological databases.

## Appendix A

**Table A1:** Prevalence per farm of Apicomplexans parasites in Zapotillo Canton, Ecuador.

Farm (*n*)	Feces (*n*)	Sera (*n*)	*N. caninum*	*T. gondii*	*Cryptosporidium* spp.	*Eimeria* spp.
1	25	11	9.09	9.09	16.00	96.00
2	17	19	10.53	10.52	5.88	58.82
3	26	18	22.22	61.11	3.85	100.00
4	25	12	0.00	0.00	8.00	72.00
5	17	16	81.25	0.00	5.88	100.00
6	15	15	64.29	13.33	20.00	100.00
7	15	18	33.33	27.77	12.33	86.67
8	18	16	43.75	18.75	16.67	94.44
9	19	16	12.50	0.00	5.26	89.47
10	15	16	0.00	12.50	0.00	80.00
11	16	15	0.00	26.66	12.50	100.00
12	15	14	0.00	14.29	13.33	100.00
13	17	14	0.00	14.29	5.88	100.00
14	14	13	0.00	0.00	14.29	92.86
15	15	14	0.00	14.29	6.67	93.33
16	15	15	0.00	33.33	6.67	100.00
17	18	16	0.00	50.00	16.67	88.89
18	17	17	0.00	29.41	35.29	94.12
19	18	18	0.00	6.25	5.56	83.33
20	18	19	10.53	10.53	16.67	66.67
21	18	19	0.00	10.53	5.56	94.44
22	18	19	0.00	16.66	0.00	83.33
23	N/A	20	5.00	18.18	N/A	N/A
24	N/A	18	0.00	25.00	N/A	N/A

**Table A2:** Univariable analysis of variables associated with *Neospora caninum* and *Toxoplasma gondii* infections in goats from Zapotillo Canton, Ecuador.

Variable	Category	*Neospora caninum*				*Toxoplasma gondii*			
		Pos (*n*)	Neg (*n*)	Chi^2^	*p*-Value	Pos (*n*)	Neg (*n*)	Chi^2^	*p*-Value
Main activity	Agriculture	1	74	10.15	0.001	--	--	--	--
	Cattle raising	30	140	Ref		--	--	--	
Total area	<15 ha	17	201	8.70	0.003	19	133	4.75	0.029
	>15 ha	32	157	Ref		46	170	Ref	
Grazing area	<15 ha	15	184	8.03	0.005	21	150	6.36	0.012
	>15 ha	43	225	Ref		44	153	Ref	
Grazing area	Known	4	116	12.58	<0.001	--	--	--	--
	Unknown	20	97	Ref		--	--	--	
Management	Intensive	27	244	3.9	0.048	--	--	--	--
	Extensive	1	74	Ref		--	--	--	
Body condition	Good	45	278	5.99	0.014	--	--	--	--
	Regular	2	63	Ref		--	--	--	
Dairy farm	Yes	--	--	--	--	12	25	6.17	0.013
	No	--	--	--		53	278	Ref	
Dual purpose	Yes	--	--	--	--	52	268	3.37	0.066
	No	--	--	--		13	35	Ref	
% of kids/total	<22%	--	--	--	--	40	137	5.71	0.017
	>22%	--	--	--		25	166	Ref	
Irrigation	Yes	6	93	4.57	0.032	20	63	3.05	0.081
	No	41	248	Ref		45	240	Ref	
Presence of artiodactyls	Yes	3	113	14.11	<0.001	23	82	1.82	0.178
	No	44	228	Ref		42	221	Ref	
Presence of cattle	Yes	1	89	13.32	<0.001	--	--	--	--
	No	46	252	Ref		--	--	--	
Presence of cats	Yes	13	152	4.84	0.028	--	--	--	--
	No	34	189	Ref		--	--	--	
Domestic fowl	Yes	43	209	16.55	<0.001	48	197	1.88	0.171
	No	4	132	Ref		17	106	Ref	
Facilities	Yes	46	271	9.35	0.002	--	--	--	--
	No	1	70	Ref		--	--	--	
Ventilation	Yes	47	308	4.97	0.026	--	--	--	--
	No	0	33	Ref		--	--	--	
Frequency of cleaning	<3 months	36	156	15.73	<0.001	--	--	--	--
	>3 months	11	185	Ref		--	--	--	
Water troughs	Yes	18	223	12.88	<0.001	47	192	1.88	0.170
	No	29	118	Ref		18	111	Ref	
Type of water	Puddled	14	111	25.10	<0.001	--	--	--	--
	Running	33	230	Ref		--	--	--	
Origin of water	Natural	29	97	20.83	<0.001	14	101	3.47	0.063
	Catchment	18	244	Ref		51	202	Ref	
Type of water troughs	Plastic	4	88	6.83	0.009	--	--	--	--
	Cement	43	253	Ref		--	--	--	
Food supplementation	Yes	10	113	2.68	0.101	27	94	2.68	0.102
	No	37	228	Ref		38	209	Ref	
Supplementation with minerals/vitamins	Yes	44	165	34.00	<0.001	--	--	--	--
	No	3	176	Ref		--	--	--	
Troughs	Yes	--	--	--	--	35	107	7.76	0.005
	No	--	--	--		30	196	Ref	
Tire troughs	Yes	0	30	4.48	0.034	9	21	3.42	0.064
	No	47	311	Ref		56	282	Ref	
Cement troughs	Yes	34	91	39.43	< 0.001	--	--	--	--
	No	13	250	Ref		--	--	--	
Type of pasture	Natural	--	--	--	--	54	285	8.89	0.003
	Cultivated	--	--	--		11	18	Ref	
Pasture fertilization	Yes	11	36	6.40	0.011	16	31	9.94	0.002
	No	36	305	Ref		49	272	Ref	
Ectoparasites	Yes	11	170	11.61	0.001	--	--	--	--
	No	36	171	Ref		--	--	--	
Infection with louses	Yes	0	82	14.33	<0.001	24	58	9.77	0.002
	No	47	259	Ref		41	245	Ref	
Application of sulfas	Yes	--	--	--	--	30	81	9.58	0.002
	No	--	--	--		35	222	Ref	
Deworming	Yes	23	118	3.67	0.055	32	109	3.98	0.046
	No	24	223	Ref		33	194	Ref	
Frequency of deworming	Regular	42	106	59.46	<0.001	--	--	--	--
	Irregular	5	235	Ref		--	--	--	
Duration of diarrhoea	<3 days	25	239	5.42	0.020	49	199	2.30	0.130
	>3 days	22	102	Ref		16	104	Ref	
Age of diarrhoea	<30 days	24	294	34.52	<0.001	60	254	3.073	0.080
	>30 days	23	47	Ref		5	49	Ref	
Vaccination	Yes	--	--	--	--	13	35	3.37	0.066
	No	--	--	--		52	268	Ref	
Milk production	<770 mL	7	184	25.22	<0.001	--	--	--	--
	>770 mL	40	157	Ref		--	--	--	
Abortions	Yes	40	319	4.26	0.039	--	--	--	--
	No	7	22	Ref		--	--	--	
Dogs’ consumption of abortion products	Yes	0	43	6.67	0.010	--	--	--	--
	No	47	298	Ref		--	--	--	--
Abortions kept in the pasture	Yes	9	214	32.14	<0.001	42	161	2.85	0.091
	No	98	127	Ref		23	142	Ref	
*Eimeria* spp. prevalence	Yes	26	181	2.47	0.116	--	--	--	--
	No	1	1	Ref		--	--	--	
Technical Visits	Yes	38	187	11.47	0.001	--	--	--	--
	No	9	154	Ref		--	--	--	

Pos positive; Neg negative; Ref reference category.

**Table A3:** Univariable analysis of variables associated with *Cryptosporidium* spp./*Eimeria* spp. infections in goats from Zapotillo Canton, Ecuador.

Variable	Category	*Cryptosporidium* spp.				*Eimeria* spp.			
		Pos (*n*)	Neg *(n*)	Chi^2^	*p*-value	Pos (*n*)	Neg (*n*)	Chi^2^	*p*-Value
Age group	<6 m	27	133	11.78	0.001	152	8	8.68	0.003
	>6 m	14	217	Ref		198	33	Ref	
Body condition	Good	28	297	7.18	0.007	--	--	--	--
	Regular	13	53	Ref		--	--	--	
Famacha	No dose	--	--	--	--	81	14	2.42	0.120
	Dose	--	--	--		269	27	Ref	
Dairy farm	Yes	2	41	1.75	0.186	36	7	1.73	0.189
	No	39	309	Ref		314	34	Ref	
Irrigation	Yes	7	93	1.75	0.187	93	7	1.74	0.187
	No	34	257	Ref		257	34	Ref	
Presence of cattle	Yes	--	--	--	--	87	14	1.65	0.199
	No	--	--	--		263	27	Ref	
Presence of cats	Yes	10	137	3.41	0.065	126	21	3.62	0.057
	No	31	213	Ref		224	20	Ref	
Facilities	Yes	40	318	2.13	0.144	--	--	--	--
	No	1	32	Ref		--	--	--	
Ventilation	Yes	40	318	2.84	0.092	--	--	--	--
	No	1	32	Ref		--	--	--	
Frequency of cleaning	<3 m	--	--	--	--	152	8	8.68	0.003
	>3 m	--	--	--		198	33	Ref	
Water troughs	Yes	--	--	--	--	246	34	2.89	0.089
	No	--	--	--		104	7	Ref	
Type of water troughs	Plastic	--	--	--	--	70	18	12.02	0.001
	Cement	--	--	--		280	23	Ref	
Food supplementation	Yes	--	--	--	--	117	9	2.21	0.137
	No	--	--	--		233	32	Ref	
Tire troughs	Yes	--	--	--	--	31	0	3.94	0.047
	No	--	--	--		319	41	Ref	
Type of pasture	Natural	--	--	--	--	300	40	4.54	0.033
	Cultivated	--	--	--		50	1	Ref	
Pasture fertilization	Yes	--	--	--	--	63	3	2.96	0.084
	No	--	--	--		287	38	Ref	
Infection with louses	Yes	13	71	2.84	0.092	80	4	3.73	0.053
	No	28	279	Ref		270	37	Ref	
Application of sulfas	Yes	--	--	--	--	128	8	4.71	0.030
	No	--	--	--		222	33	Ref	
Milk production	<770 mL	--	--	--	--	191	30	5.17	0.023
	>770 mL	--	--	--		159	11	Ref	
% of abortions/total females	<10%	--	--	--	--	316	25	28.27	< 0.001
	>10%	--	--	--		34	16	Ref	
Dogs consumption of abortion products	Yes	--	--	--	--	45	1	3.84	0.050
	No	--	--	--		305	40	Ref	
Abortions kept in the pasture	Yes	17	189	2.31	0.128	177	29	5.98	0.014
	No	24	161	Ref		173	12	Ref	
Technical Visits	Yes	18	206	3.35	0.067	--	--	--	--
	No	23	144	Ref		--	--	--	

Pos positive; Neg negative; Ref reference category.
