# Supplementary material for: Apicomplexans in Goat: Prevalence of Neospora caninum, Toxoplasma gondii, Cryptosporidium spp., Eimeria spp. and Risk Factors in Farms from Ecuador
Source: Animals (Basel). 2022 Aug 29;12(17):2224. doi: 10.3390/ani12172224 (PMC9454992; doi:10.3390/ani12172224)
Supplement: Supplementary file 1 [file animals-12-02224-s001.zip › animals-1796602-supplementary.pdf]

Table S1. Frequency of categorical variables recorded in coprological and serological data bases.

Variable	Categories	Coprological samples		Sera samples	
		Goats (*n*)	Frequency (%)	Goats (*n*)	Frequency (%)
Parish	Garza Real	108	27.6	90	23.2
	Zapotillo	172	44.0	167	43.0
	Cazaderos	64	16.4	81	20.9
	Limones	30	7.7	36	9.3
	Paletillas	17	4.3	14	3.6
Farm aptitude	Dairy	43	11.0	37	9.5
	Meat	25	6.4	11	2.8
	Double purpose	323	82.6	340	87.6
Main activity	Agriculture	302	77.2	313	80.7
	Breeding	89	22.8	75	19.3
Managment	Intensive	25	6.4	52	13.4
	Extensive	281	71.9	257	66.2
	Mixed	85	21.7	79	20.4
Grazing area	Known	268	68.5	268	69.1
	Unknown	123	31.5	120	30.9
Common land	Yes	366	93.6	377	97.2
	No	25	6.4	11	2.8
Irrigation	Yes	100	25.6	99	25.5
	No	291	74.4	289	74.5
Facilities	Yes	358	91.6	317	81.7
	No	33	8.4	71	18.3
Ventilation	Yes	358	91.6	355	91.5
	No	33	8.4	33	8.5
Cleaning	Yes	345	88.2	345	88.9
	No	46	11.8	43	11.1
Cleaning frequency	< 6 months	114	29.1	176	45.3
	≥ 6 months	196	50.1	169	43.6
	Never	35	9.1	43	11.1

Foot baths	Yes	25	6.4	11	2.8
	No	366	93.6	377	97.2
Drinking troughs	Yes	280	71.6	241	62.1
	No	111	28.4	147	37.9
Type of drinking trough	N/A	111	28.4	147	37.9
	Cement	192	49.1	149	38.4
	Plastic	88	22.5	92	23.7
Source of water	Well	135	34.5	167	43
	Creek	15	3.8	14	3.6
	Public system	25	6.4	11	2.8
	Spring water	86	22.0	74	19.1
	Waterways	26	6.6	18	4.6
	Chlorinated water	104	26.6	104	26.8
Feeding trough	Yes	169	43.2	142	36.6
	No	222	56.8	246	63.4
Food suplementation	Yes	126	32.2	123	31.7
	No	265	67.8	265	68.3
Vitamin suplementation	Yes	226	57.8	209	53.9
	No	165	42.2	179	46.1
Mineral suplementation	Yes	197	50.4	158	40.7
	No	194	49.6	230	59.3
Grazing	Si	366	93.6	357	92.0
	No	25	6.4	31	8.0
Types of grass	Natural	340	87.0	359	92.5
	Cultivated	51	13.0	29	7.5
Deworming	Yes	187	47.8	160	41.2
	No	204	52.2	228	58.8
Frequency of deworming	Bimonthly	68	17.4	148	38.1
	Three times/year	48	12.3	240	61.9
	Unplanned	126	32.2	48	12.4
	Unknown	149	38.1	67	17.3

Duration of deworming	Unknown	117	29.9	144	37.1
	One dose	274	70.1	129	33.3
Use of sulfa drugs	Yes	136	34.8	116	29.9
	No	255	65.2	272	70.1
Ectoparasites	Yes	187	47.8	111	28.6
	No	204	52.2	277	71.4
Vaccinations	Yes	68	17.4	181	46.6
	No	323	82.5	207	53.4
Presence of diarrheas	Yes	345	88.2	48	12.4
	No	46	11.8	340	87.6
Abortions	Yes	351	89.8	359	92.5
	No	40	10.2	29	7.5
Abortions (%) / total females	< 10%	341	87.2	..	..
	≥ 10%	50	12.8	..	..
Disposal of abortion products	Buried	..	..	47	12.1
	Throw away	..	..	11	2.8
	Burned	..	..	14	3.6
	Remains in the field	..	..	223	57.5
	Food for dogs	..	..	62	16.0
	Unknown	..	..	31	8.0
Consumption of goat meat	Yes	..	..	377	97.2
	No	..	..	11	2.8
Days of diarrhea	N/A	46	11.8	45	11.6
	< 3 days	223	57.0	219	56.4
	> 3 days	122	31.2	124	32.0
Age of diarrheas	N/A	46	11.8	45	11.6
	< 30 days	295	75.4	273	70.4
	> 30 days	50	12.8	70	18.0
Body condition	Good	325	83.1	323	83.2
	Regular	66	16.9	65	16.8
	Bad	0	0.0	0	0.0
Technical visits	Yes	224	42.7	225	58.0

	No	167	57.3	163	42.0
Presence of wild artyodactils	Yes	391	100.0	194	50.0
	No	0	0.0	194	50.0
Presence of cattle	Yes	101	25.8	90	23.2
	No	290	74.2	298	76.8
Presence of dogs	Yes	--	--	377	97.2
	No	--	--	11	2.8
Presence of domestic fowl	Yes	281	71.9	377	97.2
	No	110	28.1	11	2.8
Wild birds	Yes	391	100.0	252	64.9
	No	0	0.0	136	35.1
Phenotype	Cross-breed	316	80.8	329	84.8
	Boer	15	3.8	9	2.3
	Cross-breed x Nubian	41	10.5	36	9.3
	Cross-breed x Saanen	15	3.8	11	2.8
	Cross-breed x Alpine	4	1.0	3	0.8
Sex	Male	41	10.5	17	4.4
	Females	350	89.5	371	95.6
Age group	< 6 month	160	40.9	..	..
	> 6 month	231	59.1	..	..
Famacha	2	95	24.3	103	26.5
	3	225	57.5	222	57.2
	4	67	17.1	59	15.2
	5	3	0.8	3	0.8
Positivity to *Neospora caninum*	..	..	Seropositive	47	87.9
	..	..	Seronegative	341	12.1
Positivity to *Toxoplasma gondii*	..	..	Seropositive	67	18.0
	..	..	Seronegative	301	82.0
Positivity to *Eimeria* spp.	Yes	350	89.5	..	..
	No	41	10.5	..	..
Positivity to *Cryptosporidium* spp.	Yes	41	10.5	..	..
	No	350	89.5	..	..
